# GM-CSF promotes pro-inflammatory macrophage activation associated with Akt/mTOR signaling during experimental colitis

**DOI:** 10.3389/fimmu.2026.1799536

**Published:** 2026-06-26

**Authors:** Silan Shen, Kexin Chen, Lili Li, Mingshan Jiang, Yongbin Jia, Xiufeng Bai, Zhen Zeng, Chunxiang Ma, Yuan Dang, Kehan Hu, Yanqiong Chen, Wenting Zhang, Zhiyong Miao, Linlin Chen, Hu Zhang

**Affiliations:** 1 Department of Gastroenterology, West China Hospital, Sichuan University, Chengdu, China; 2 Centre for Inflammatory Bowel Disease, West China Hospital, Sichuan University, Chengdu, China; 3 Laboratory of Inflammatory bowel disease, Institute of Immunology and Inflammation, Frontiers Science Center for Disease-Related Molecular Network, West China Hospital, Sichuan University, Chengdu, China; 4 Laboratory of Human Disease and Immunotherapies, West China Hospital, Sichuan University, Chengdu, China; 5 Institute of Immunology and Inflammation, Frontiers Science Center for Disease-related Molecular Network, West China Hospital, Sichuan University, Chengdu, China; 6 Department of Gastroenterology, Suining Central Hospital, Suining, Sichuan, China

**Keywords:** Akt, glycolysis, granulocyte macrophage-colony stimulating factor, macrophages, mTOR, ulcerative colitis

## Abstract

**Background:**

Ulcerative colitis (UC) is an intestinal immune disorder of unknown etiology. Mounting evidence reveals a central role of macrophages in the hemostatic balance of gut immunity, and dysfunctional macrophages are associated with UC pathogenesis. Granulocyte macrophage-colony stimulating factor (GM-CSF) is an essential modulator of macrophages and has recently been recognized as a potential target in many autoimmune disorders. However, the action of GM-CSF in gut inflammation remains unspecified.

**Methods:**

The significance of GM-CSF in UC and its mechanism of action were investigated. GM-CSF expression was examined in colon biopsy tissues from UC patients and healthy controls. A dextran sodium sulfate (DSS)-induced colitis mouse model was used to evaluate the effect of GM-CSF neutralizing antibody (GM-CSF Ab). Macrophage infiltration, CD4+ T helper (Th) cell responses, macrophage polarization, and glycolysis-related genes were assessed using in vivo and in vitro experiments. The involvement of the Akt/mTOR pathway was also examined.

**Results:**

GM-CSF expression was significantly elevated in colon biopsy tissues from UC patients compared to controls. Administration of GM-CSF Ab to DSS-treated mice attenuated gut inflammation. Furthermore, GM-CSF Ab inhibited the infiltration of macrophages and inflammatory CD4+ Th cells into the intestine of DSS-colitis mice. In vitro experiments showed that GM-CSF induced M1-type polarization of peritoneal macrophages and subsequently augmented the Th17 response. Further experiments indicated that the proinflammatory phenotype of macrophages induced by GM-CSF was related to glycolytic metabolism, which was influenced by the Akt/mTOR pathway.

**Conclusion:**

Collectively, this study suggests that GM-CSF is associated with the regulation of glycolytic metabolism involving the Akt/mTOR pathway, and subsequently alters macrophage function to promote intestinal inflammation.

## 1 Introduction

Ulcerative colitis (UC) is an inflammatory disorder that occurs in the colon and rectum, with a chronic and recurrent course (1–3). Over the last decades, the incidence and prevalence of UC have risen, giving rise to a high global burden (1, 4–6). Despite grave progress in our understanding of UC pathogenesis, the therapeutic strategies for UC are still unsatisfactory and are often involved in inevitable side effects (7–12). Therefore, further exploration of UC pathogenesis for improving treatments is essential.

Although the onset of UC was previously attributed to abnormal intestinal T-cell responses, emerging studies have indicated the contribution of dysfunctional innate immune cells to the pathogenesis of UC (13–17). Macrophages, part of innate immunity, play as a central coordinator in the intestinal immune cellular network, with the ability to respond to microenvironmental stimuli and mediate immune tolerance or inflammation (18–21). Macrophages show strong plasticity, which allows them to shape into different phenotypes upon exposure to environmental signals (22). Traditionally, macrophages are divided into two phenotypes depending on their physical functions (23). One is classically activated (M1) proinflammatory macrophages, which are generally stimulated by LPS and IFN-γ and then elevate the production of inflammation-related cytokines, typically IL-1β, TNF-α and IL-6 (24, 25). The M1 phenotype is often characterized by inducible nitric oxide synthase (iNOS/NOS2) as well as the cell surface protein CD86. The other is alternatively activated (M2) anti-inflammatory macrophages, which are marked by arginase-1 (Arg-1), typically driven by IL-4 and IL-13 to polarize and then produce anti-inflammatory cytokines, such as IL-10 (15, 24, 25). Overactivated proinflammatory macrophages are related to the onset and relapse of UC (26). Recent studies have further elucidated the metabolic and signaling pathways governing macrophage polarization in UC, highlighting the potential of targeting these pathways for therapeutic intervention (27, 28). However, the factors and pathways modulating macrophage responses during intestinal inflammation are still largely obscure.

Granulocyte macrophage-colony stimulating factor (GM-CSF) is a considerable factor that regulates innate immune cells functions and mediates the link between innate immunity and adaptive immunity. GM-CSF has been highlighted in inflammatory disorders recently (29). It was primitively regarded as a myelopoietic growth factor because of its ability to form bone marrow hematopoietic progenitor colonies and differentiate them into granulocyte and macrophage colonies (30). Later, studies revealed that GM-CSF functioned as a cytokine via the receptor, regulating the proliferation, survival, activation and differentiation of myeloid cells, among which macrophages were a major target (31). A number of reports have illustrated that GM-CSF is related to many inflammatory diseases (32–36). Due to the inflammatory properties of GM-CSF, therapeutic strategies targeting GM-CSF have been created, and clinical trials have implied the efficacy of these treatments (32, 37, 38). Nevertheless, GM-CSF was beneficial in some autoimmune settings, such as autoimmune diabetes and type 2 diabetes (39, 40). Abundant studies have indicated the dual role of GM-CSF in inflammation (41). Reports have suggested that GM-CSF promotes intestinal inflammation by activating eosinophils and recruiting inflammatory monocytes (42, 43). However, some studies have suggested a protective function of GM-CSF in intestinal inflammation because it can promote epithelial repair and inhibit inflammation (44–46). These contradictions raised questions about the precise effects of GM-CSF on UC pathogenesis and how GM-CSF modulates macrophages to coordinate gut inflammation.

Here, our study confirmed the upregulation of GM-CSF in UC patients. Administration of GM-CSF Ab to DSS-induced colitis mice ameliorated intestinal inflammation, which suggested the proinflammatory effect of GM-CSF in colitis. Mechanistically, integrating recent advances in immune metabolism (47, 48), we further explored the latent mechanism by which GM-CSF promoted the inflammatory responses of macrophages. This work indicated that GM-CSF might trigger the Akt/mTOR pathway to modulate glucose metabolism in macrophages and then induce macrophages to polarize into the M1 type and aggravate intestinal inflammation.

## 2 Materials and methods

### 2.1 Human colonic biopsy specimens

This study comprised 22 patients who had undergone colonoscopy at West China Hospital. The case group contained 11 patients with UC, and mucosal specimens were harvested from inflamed areas of colons, while the control group included 11 patients with colonic polyps, and control colonoscopic biopsies were taken from normal colonic areas. The study protocol was approved by the Clinical Ethics Committee of West China Hospital of Sichuan University (Reference numbers: 2019-970).

### 2.2 Immunofluorescence

The expression of GM-CSF in colonic biopsy specimens was evaluated by immunofluorescence. Paraffin specimens were minced into 5-μm-thick fragments and adhered to adhesion microscope slides. After pretreatment, sections were incubated with GM-CSF antibody (17762-1-AP, Proteintech) at 4 °C overnight. Sections were washed with phosphate-buffered saline (PBS) and distilled water successively. Then, the sections were incubated at room temperature for 45 min with Alexa 488-conjugated secondary antibody (4412, CST). After washed with PBS and distilled water, the sections were stained with DAPI. The sections were washed again and sealed with Anti-Fade Fluorescence Mounting Medium (ab104135, Abcam) after washed with PBS and distilled water. Sections were observed and photographed under a fluorescence microscopy (Carl Zeiss). The images were analyzed by ImageJ software. Immunofluorescence was also used to assess the infiltration of macrophages in mouse colons. An F4/80 antibody (29414-1-AP, Proteintech) was applied to identify macrophages in mouse colons.

### 2.3 Mice models

Male C57/BL/6J mice (20-30g), purchased from Huafukang (Beijing, China), were raised in specific pathogen-free (SPF) conditions. To induce acute colitis, mice were exposed to 2.5% (*w/v*) DSS (9011-18-1, MP Biomedicals) in drinking water for 7 days. Then, these colitis mice were treated with InVivoMAb anti-mouse GM-CSF (MP1-22E9, BioXCell) (GM-CSF Ab) at doses of 100μg/mouse (100μl, diluted with PBS) or PBS 100μl/mouse via intraperitoneal injection every other day. Furthermore, mice without colitis were also treated with 100μl PBS per mouse via intraperitoneal injection every other day to be negative controls. The three groups were named CTRL, DSS+PBS, and DSS+GM-CSF Ab. The mice were sacrificed for tissue sampling on the eighth day. The spleens were weighed immediately after being separated from the sacrificed mice. The entire colons were also excised from the mice. The colon length was measured from the ileocecal junction to the anus. Subsequently, the colon tissues were processed for RNA extraction, protein extraction, histology and flow cytometric analysis.

Animal experiments were approved by the Institutional Animal Care and Use Committees (IACUC) of West China Hospital, Sichuan University (Reference numbers: 2019297A).

### 2.4 Disease activity index and spleen index

The DAI of colitis mice was defined by total scores of body weight loss, stool consistency and intestinal bleeding. The general physical conditions of the mice were monitored and recorded daily. Body weight loss scores were defined as follows: 0, no loss; 1, 1-5%; 2, 5-10%; 3, 10-20%; 4, >20%. Scores of stool consistency were determined as follows: 0, normal; 1, soft but still formed; 2, soft; 3, very soft and wet; 4, watery diarrhea. Scores of gross bleedings were defined as follows: 0, no blood; 2, visible blood in stool; 4, rectal bleeding (49, 50).

On the last day, the spleen index was measured to assess immune function alterations. The spleen index was calculated as spleen weight (mg)/body weight (1000mg) (51).

### 2.5 Histological assessment

Colon samples were fixed in 4% paraformaldehyde and embedded in paraffin, followed by sectioning at 5 µm thickness and sticking to slides. After hematoxylin and eosin (HE) staining, histological assessment was performed, which was based on tissue damage, inflammation extent and depth of inflammatory infiltration. Tissue damage was scored according to the fraction of lesion range: 0, normal; 1, <10%; 2, 10-25%; 3, 25-50%; 4, >50%. The extent of inflammation was graded according to the proportion of affected tissue: 0, normal; 1, <10%; 2, 10-25%; 3, 25-50%; 4, >50%. The depth of inflammation infiltration scoring was defined as follows: 0, absent; 1, around the crypt; 2, muscularis mucosa; 3, submucosa; 4, muscularis and serosa (52, 53). The total histological score was obtained by adding the scores of the three sections, ranging from 0 to 12 points. Histological scoring was performed in a blinded manner.

### 2.6 Isolation of lamina propria mononuclear cells

To isolate LPMCs, colons were opened longitudinally and cut into 0.5-1cm pieces after washed thoroughly with ice-cold PBS. The tissue pieces were shaken for 20 min at 37 °C in 10 ml D-HBSS containing 1 mM DTT, 5 mM EDTA and 5% FCS. These tissues were flushed and minced. Then, tissue pieces were shaken for 30 min at 37 °C in 5 ml of HBSS with 5% FCS, 1 mg/ml type IV collagenase (C8160, Solarbio) and 2mg/ml DNase I (D8071, Solarbio). Cell solutions were passed through 70-μm strainers to filter single cells from the remaining tissues. Later, these single cells were washed with PBS containing 2.5% FCS. The cells were resuspended in RPMI 1640 containing 1% penicillin–streptomycin liquid and 10% FCS for further treatment.

### 2.7 Flow cytometry

Single cells were stimulated with Leuko Act Cktl with GolgiPlug (550583, BD) for 4-6h at 37 °C in an incubator with 5% CO2. Cells were collected and incubated with Fixable Viability Stain 780 (565388, BD) for 10 min at room temperature. Next, Ms CD16/CD32 (553141, BD) was used to block the cells at 4 °C for 10 min. Surface protein staining of cells was performed at 4 °C for 30 min. Cells were incubated with the following antibodies: Ms CD45 Alexa 700 (560510, BD), Ms CD3e BUV395 (563565, BD), and Ms CD4 BV510 (563106, BD). For intracellular protein staining, cells were first treated with the Foxp3 staining kit (00-5523-00, Thermo Fisher) and then stained with the following antibodies at 4 °C for 45 min: Ms IFN-Gma BV711 (564336, BD), Ms IL-17A BV421 (563354, BD), and Ms Foxp3 PE (560408, BD). All antibodies were diluted 1:100 in PBS. Cells were counted using a FACSAria SORP (BD, USA), and FlowJo software (version 10) was applied to analyze the data. The gating strategy for flow cytometry is detailed in **Supplementary Text S1**.

### 2.8 Quantitative real-time PCR

RNAs were extracted from mouse colonic tissues or cells using TRIzol^®^ Reagent (15596026, Invitrogen). Total RNAs were reverse transcribed to cDNA using a FastKing One Step RT–PCR Kit (KR123, TIANGEN). Quantitative real-time PCR was performed using 2×T5 Fast qPCR Mix (SYBR Green I) (TSE202, Tsingke Biological Technology Co., Ltd.). The specific primers used in the study for mice were as follows: *Actb* (5’-GTGACGTTGACATCCGTAAAGA-3’ and 5’-GCCGGACTCATCGTACTCC-3’), *Il6* (5’-AGCCAGAGTCCTTCAGAGAGATAC-3’ and 5’-AATTGGATGGTCTTGGTCCTTAGC-3’), *Tnf* (5’-ATGGCCTCCCTCTCATCAGT-3’ and 5’-TTTGCTACGACGTGGGCTAC-3’), *Il1* (5’-GCAACTGTTCCTGAACTCACT-3’ and 5’-ATCTTTTGGGGTCCGTCAACTC-3), *Nos2* (5’-GTTCGCCCAACAATACAAGA-3’ and 5’-GTGGACGGGTCGATGTCAC-3), *Il10* (5’-AGCCTTATCGGAAATGATCCAGT-3’ and 5’-GGCCTTGTAGACACCTTGGT-3’), *Arg1* (5’-ATGCTCACACTGACATCAACACTC-3’ and 5’-CTCTTCCATCACCTTGCCAATCC-3’), *Hif1a* (5’-TCTCGGCGAAGCAAAGGTC-3’ and 5’-AGCCATCTAGGGGCTTTCAGATA-3), *Slc2a1* (5’-CAGTTCGGCATAACACTGGTGG-3’ and 5’-GCCCCCGACAGAGAGAGATG-3), *Hk2* (5’-TGATCGCTGCTTATTCAGG-3’ and 5’-AACCGCCTAGAATCCCAGA-3), *Pfkfb3* (5’-TCTCGGCGAAGCAAAGGTC-3’ and 5’-AGCCATCTAGGGGCTTTCAGATA-3). RNA expression was calculated based on the 2^−ΔΔCT^ method, taking the β-actin gene as the internal control.

### 2.9 Western blotting

RIPA buffer (high) (R0010, R0010) was used to lyse cells or tissues according to the instructions. A BCA protein concentration assay kit (AR1189, BOSTER) was applied to determine the protein concentrations. The antibodies used here were as follows: anti-Akt (1:1000, 9272, CST), anti-phosphorylation-Akt (1:1000, 4060, CST), anti-mTOR (1:1000, T55306, Abmart), anti-phosphorylation-mTOR (1:1000, T56571, Abmart), anti-GAPDH (1:1000, BX-008, BioX), anti-CD206 (1:1000, ab64693, Abcam), anti-iNOS (1:1000, ab178945, Abcam), and anti-α Tubulin (1:2000, 11224-1-AP, Proteintech). Protein bands were analyzed using ImageJ software, and GAPDH or α-Tubulin was used as a reference.

### 2.10 Isolation of peritoneal macrophages and purification of CD4^+^ T cells

Male C57BL/6 mice were administered 2ml of 3% thioglycollate via intraperitoneal injection for three days, and peritoneal macrophages were collected on day 4. Cells were seeded into 6-well plates at 2 × 10^6^ cells/well and cultured in RPMI 1640 containing 10% FCS and 1% penicillin–streptomycin liquid. These cells were divided into a control group and a GM-CSF group. Cells in the control group were treated with the solvent (PBS), and cells in the GM-CSF group were treated with 10ng/ml recombinant mouse GM-CSF protein (rm GM-CSF) (51048-MNAH, SinoBiological) for 48 hrs.

The spleen was immediately removed from the sacrificed mice, and a MojoSort™ Mouse CD4 T Cell Isolation Kit (480006, BioLegend) was used to isolate CD4^+^ T cells according to the instructions. Twenty-four-well plates were precoated with 1µg/ml CD3e Antibody (14-0031-82, eBioscience) and 1µg/ml CD28 Antibody (14-0281-82, eBioscience) at 4 °C overnight. Peritoneal macrophages (treated or untreated with rm GM-CSF) and CD4^+^ T cells were seeded into the pretreated 24-well plate at 2 × 10^5^ cells/well and 1 × 10^6^ cells/well, respectively. Cells were cultivated in RPMI 1640 containing 1% penicillin–streptomycin liquid and 10% FCS. After 72 hrs, the cells were obtained for further experiments.

### 2.11 Statistical analysis

Statistical analysis and graph generation were conducted by GraphPad Prism software (version 8.0.2). Data were shown as the mean ± standard error of the mean (SEM). Comparisons of two groups were assessed by Student’s t test. For multiple groups (more than two groups), the differences were analyzed by one-way ANOVA or two-way ANOVA. When significant differences were detected by one-way or two-way ANOVA, Tukey’s multiple comparisons test was used for *post-hoc* pairwise comparisons. *P* values < 0.05 were considered statistically significant.

## 3 Results

### 3.1 The expression level of GM-CSF was upregulated in inflamed colonic tissues of UC patients

**Figure 1:**
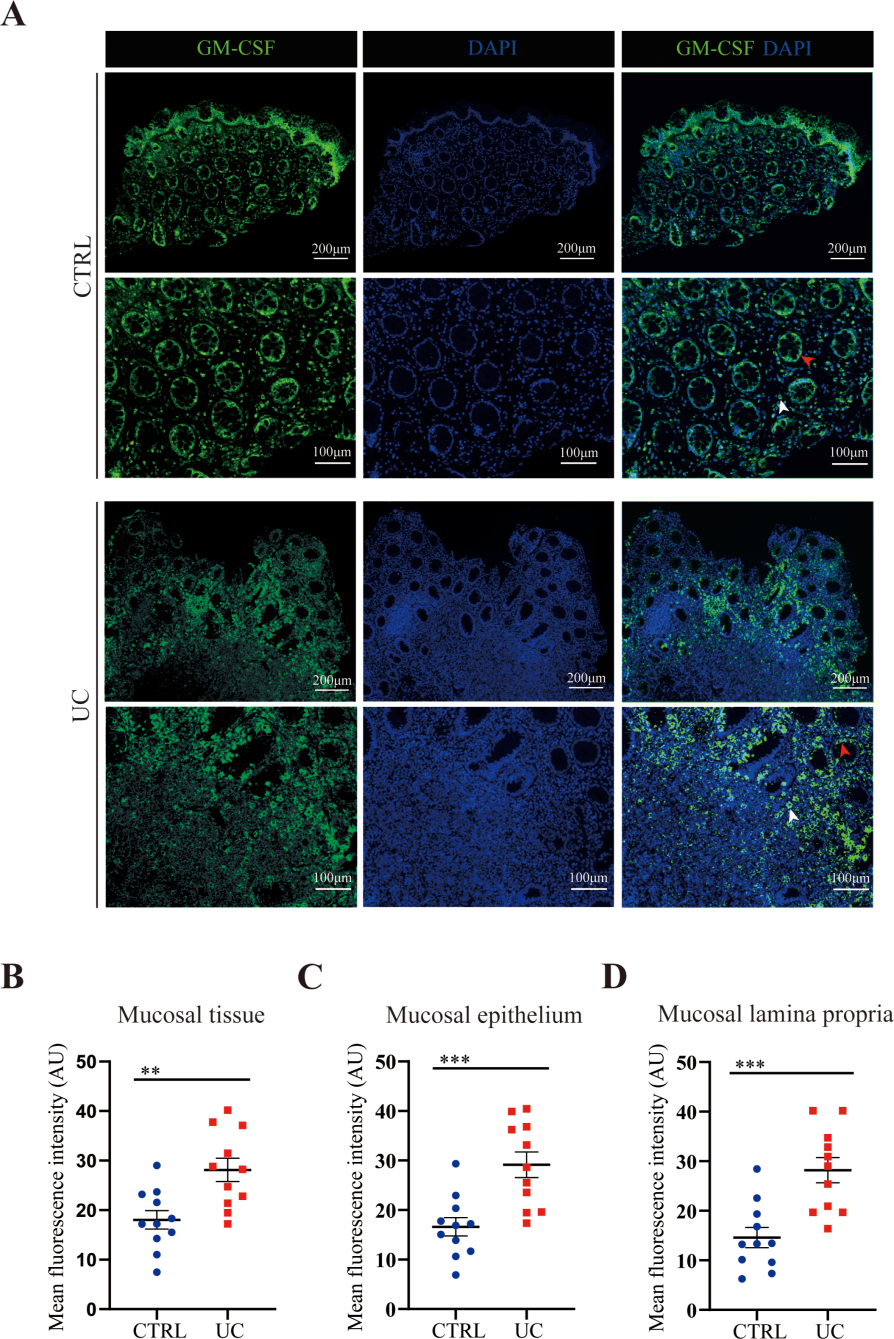
Expression of GM-CSF in colonic tissues of controls and UC patients. **(A)** Representative photomicrographs of GM-CSF-stained colonic tissue paraffin sections from controls and patients with UC (scale bar: 200μm and 100μm) (GM-CSF: Green). **(B)** Statistical analysis of the mean fluorescence intensity of GM-CSF-stained colonic tissue paraffin sections from controls and patients with UC. Statistical analysis of the mean fluorescence intensity of GM-CSF in the epithelial layer **(C)** and lamina propria **(D)** of the intestinal mucosa from controls and UC patients. Red arrow indicates GM-CSF^+^ epithelial cells, and white arrow indicates GM-CSF^+^ lamina propria cells. **P< 0.01, ***P< 0.001.

To detect the change in GM-CSF during UC, immunofluorescence for GM-CSF was executed on paraffin sections of colonic biopsy specimens from controls and UC patients. As shown in **Figure 1A**, the fluorescence intensity of GM-CSF in UC was stronger than that in the controls. For statistical analysis, the average fluorescence intensity was quantified using ImageJ software. Compared to normal controls, GM-CSF expression increased in UC patients, and the increase in UC patients was statistically significant (**Figure 1B**). Furthermore, GM-CSF is expressed both in cells of the epithelium and in cells of the lamina propria. Further analysis showed that both on the mucosal epithelium and on the mucosal lamina propria, GM-CSF expression in UC patients was notably higher than that in controls (**Figures 1C, D**). This result suggested that the increase in GM-CSF during intestinal inflammation was attributed to both mucosal epithelial cells and cells of the mucosal lamina propria.

### 3.2 Therapeutic effect of GM-CSF Ab on mice with DSS-induced colitis

**Figure 2:**
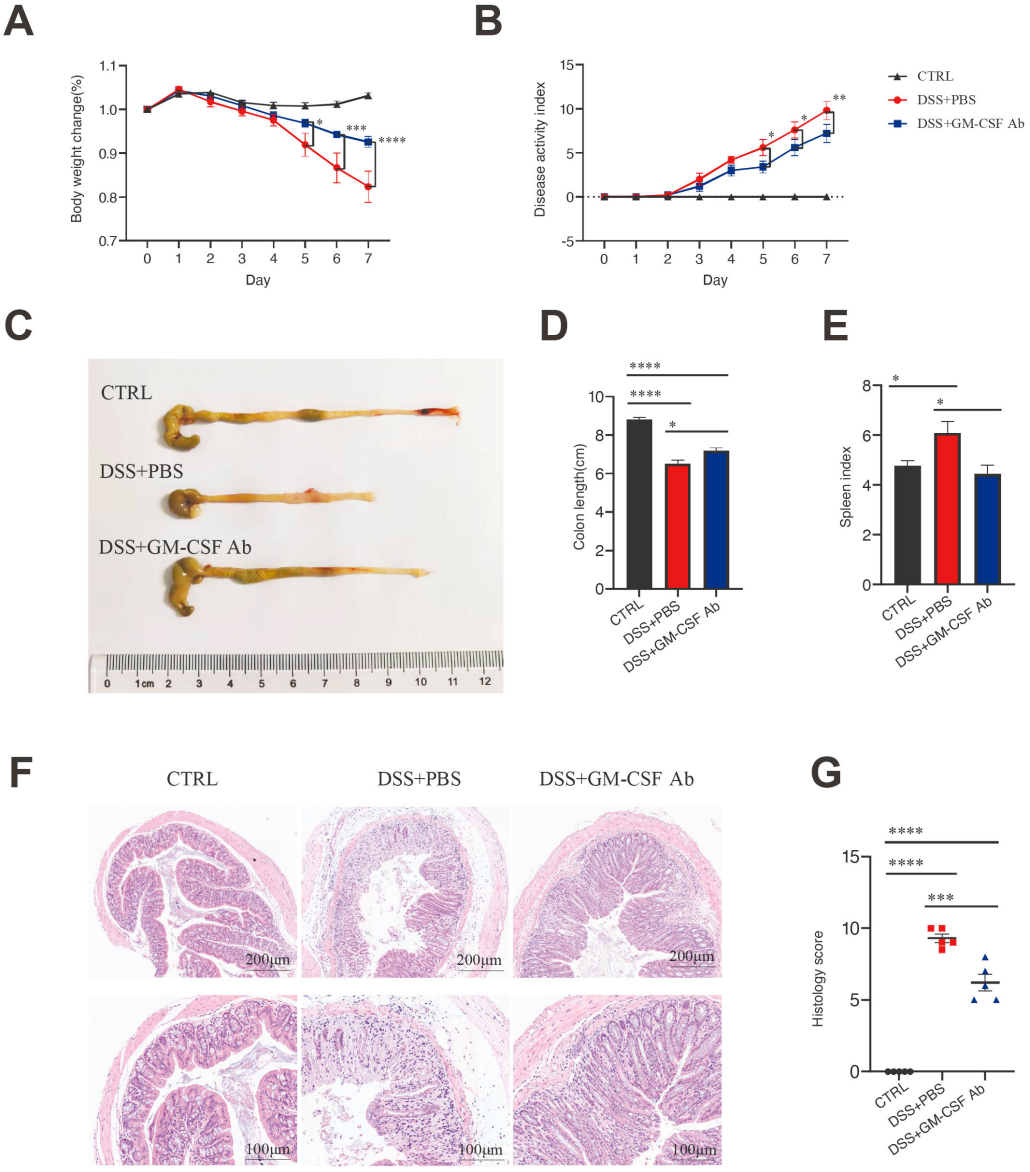
GM-CSF Ab ameliorated DSS-induced colitis in mice. **(A)** Daily body weight changes of mice. **(B)** DAI during colitis. **(C, D)** Colon lengths. **(E)** Spleen indexes. **(F)** Representative photomicrographs of HE staining of colonic tissues from the CTRL group, DSS+PBS group and DSS+GM-CSF Ab group (scale bar: 200μm and 100μm). **(G)** Histology scores of mice colonic tissues. * P< 0.05, ** P< 0.01, ***P< 0.001, ****P< 0.0001.

As the similarities to human UC in clinical and pathological manifestations, DSS-induced colitis mice were used to explore the impact of GM-CSF in UC (49). To induce colitis, 2.5% DSS solution was administered to C57BL/6 mice for 7 days. In addition, GM-CSF Ab was administered intraperitoneally every two days to block the GM-CSF signaling pathway. During DSS-induced colitis, GM-CSF Ab treatment ameliorated the intestinal inflammation of DSS-induced mice. Mice receiving GM-CSF Ab displayed remarkably lower weight loss during DSS treatment, which showed a significant difference at day 5 and was more salient at day 7 (**Figure 2A**). Consistent with changes in weight loss, DSS mice treated with GM-CSF Ab demonstrated significantly lower DAI scores from day 5 on (**Figure 2B**). Macroscopic examinations were conducted at the end of the experiment, which revealed longer colon lengths in GM-CSF Ab-treated DSS mice (**Figures 2C, D**). Compared to the DSS+PBS group, the spleen index was lower in the DSS+GM-CSF Ab group, which also implied less severe inflammation (**Figure 2E**). Besides, pathological images exhibited that mice given GM-CSF Ab had milder colonic tissue damage, a smaller inflammation scale and less inflammatory cell infiltration than those given PBS during DSS treatment (**Figure 2F**). The DSS mice with GM-CSF Ab-treatment showed obviously lower histology scores than those with PBS-treatment (**Figure 2G**). These parameters suggested the therapeutic role of GM-CSF Ab in colitis, which also prompted the opposite action of GM-CSF during colitis at the meantime.

### 3.3 GM-CSF Ab restored the balance of intestinal immune cells during colitis in mice

**Figure 3:**
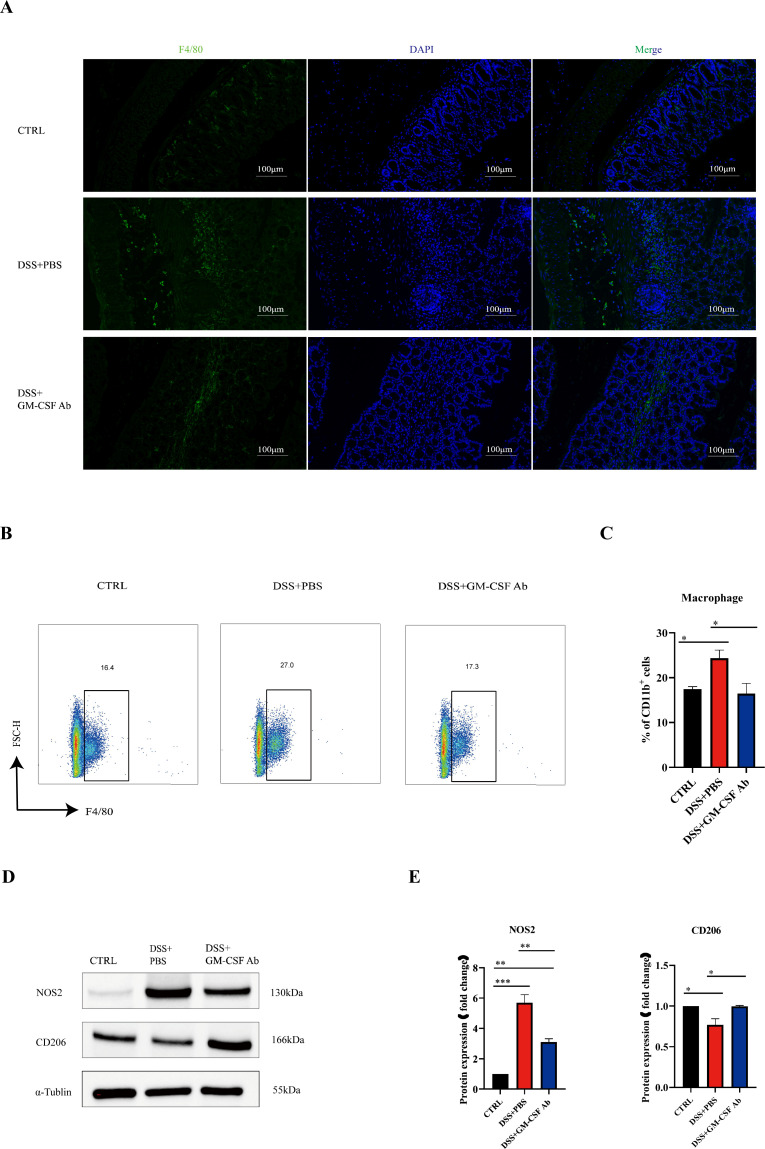
GM-CSF Ab remodeled the balance of M1/M2 macrophages in the colon of DSS-induced colitis mice. **(A)** Immunofluorescence staining of F4/80 for macrophages in colon tissues from the CTRL group, DSS+PBS group and DSS+GM-CSF Ab group. **(B, C)** The frequencies of macrophage in mice colons. **(D, E)** Protein expressions of NOS2 and CD206 of colon tissues from the CTRL group, DSS+PBS group and DSS+GM-CSF Ab group. * P< 0.05, ** P< 0.01, ***P< 0.001.

**Figure 4:**
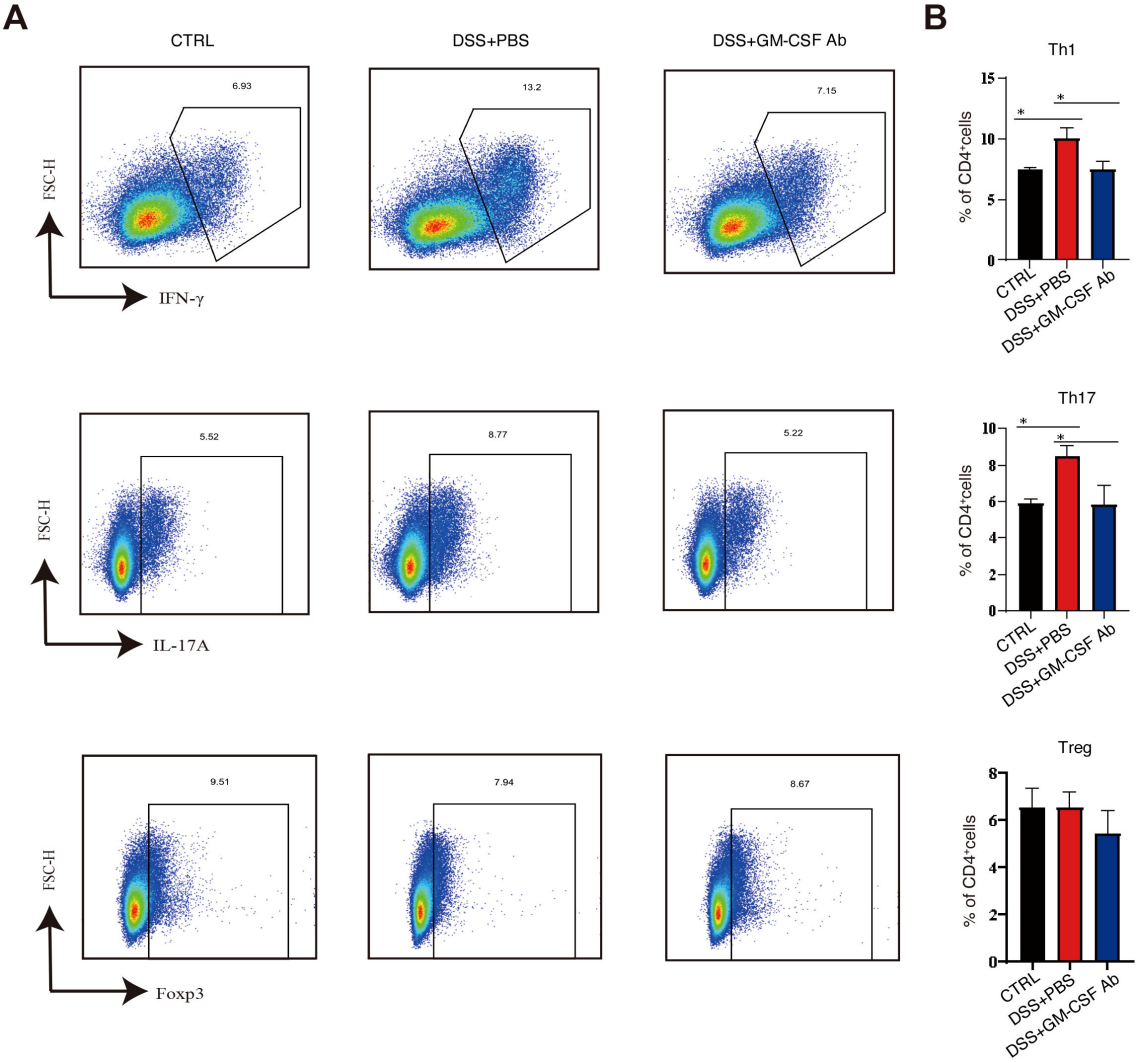
GM-CSF Ab inhibited the infiltration of proinflammatory Th cells in the colon of DSS-induced colitis mice. **(A, B)** The frequencies of Th1 (IFN-γ^+^ CD4^+^ T) cells, Th17 (IL-17A^+^ CD4^+^ T) cells and Treg (Foxp3^+^ CD4^+^ T) cells in mice colons. *P< 0.05.

Intestinal immune imbalance is a major feature of UC, especially the accumulation of proinflammatory immune cells. As the main target of GM-CSF, macrophages in the mice intestines were detected. The immunofluorescence results of macrophages (marked with F4/80) showed that DSS promoted the infiltration of macrophages into the intestine, but administering GM-CSF Ab to DSS mice inhibited the infiltration (**Figures 3A–C**). Subsequently, we explored if GM-CSF Ab regulates the balance of different macrophage phenotypes in colitis tissues, as previous studies suggested that excessive M1 macrophages and inadequate M2 macrophages resulted in exacerbated inflammation (54). We detected the expressions of inflammatory macrophage and anti-inflammatory macrophage marker proteins in mice colons. As shown in **Figure 3D** and E, GM-CSF Ab significantly decreased the level of NOS2 (M1-associated marker protein) and increased the level of CD206 (M2-associated marker protein). Given that Th cells have pivotal roles in the onset and progression of UC, the alteration of Th cells in the mice gut was investigated by flow cytometry (55). Compared to the control group, DSS administration contributed to the significant increases in IFN-γ-producing T helper (Th1) cells and IL-17A-producing T helper (Th17) cells in the mice intestines, but GM-CSF Ab altered the increases in proinflammatory Th cell subsets in DSS mice (**Figures 4A, B**). In terms of the percentage of Foxp3^+^ CD4^+^ T (Treg) cells, no significant difference was found among the three groups (**Figures 4A, B**). GM-CSF Ab was advantageous for recovering the balance of intestinal immune cells, indicating the proinflammatory action of GM-CSF during colitis.

### 3.4 GM-CSF promoted pro-inflammatory macrophage activation and was associated with enhanced Th17 responses

**Figure 5:**
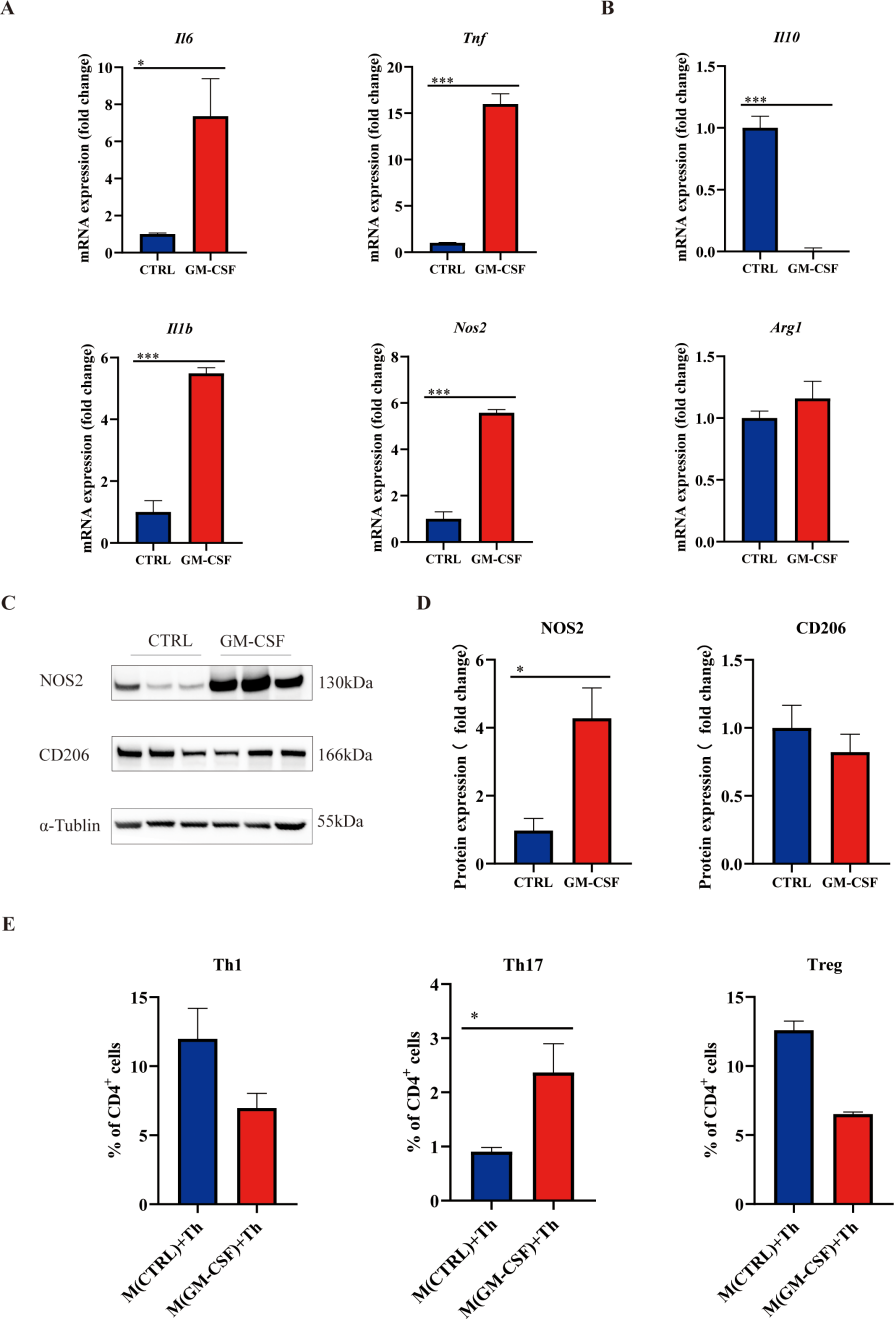
GM-CSF promoted the activation of peritoneal macrophages and regulated Th17 cell responses. Peritoneal macrophages were treated with rm GM-CSF or the solvent. **(A)** The mRNA expression levels of *Il6*, *Tnf*, *Il1b*, and *Nos2* in peritoneal macrophages. **(B)** The mRNA expression levels of *Il10* and *Arg1* in peritoneal macrophages. **(C, D)** The expression levels of NOS2 and CD206 in peritoneal macrophages. **(E)** Frequencies of Th1 (IFN-γ^+^) cells, Th17 (IL-17A^+^) cells and Treg (Foxp3^+^) cells in CD4^+^ T cells that were cocultured with peritoneal macrophages. * P< 0.05, ***P< 0.001.

Bioinformatics analysis indicated that GM-CSF was crucial for macrophage action in DSS-induced colitis (56). Resident macrophages in different tissues have specific functions shaped by the surrounding environment, and they may show different responses to the same specific signals (57). To further assess the role of GM-CSF in gut macrophages, peritoneal macrophages, which may be more representative of the responses of intestinal macrophages, were selected as model cells. rm GM-CSF was applied to stimulate peritoneal macrophages to test their responses. The M1 phenotype-associated genes, including *Il6*, *Tnf*, *Il1b* and *Nos2*, were elevated in rm GM-CSF-treated macrophages, while the mRNA expression level of *Il10*, one of the factors representing the M2 phenotype, was declined (**Figures 5A, B**). However, *Arg1*, another M2-type gene, did not change significantly (**Figure 5B**). In addition, rm GM-CSF markedly upregulated the expression of NOS2 (M1 marker protein) in peritoneal macrophages (**Figures 5C, D**). These results indicated that GM-CSF promoted macrophage polarization into the proinflammatory type.

Given the interaction of innate immunity and adaptive immunity, the influence of macrophages treated with rm GM-CSF on CD4^+^ T cells was evaluated. Macrophages treated and untreated with rm GM-CSF were cocultured with CD4^+^ T cells. CD4^+^ T cells cocultured with rm GM-CSF-treated macrophages had a significant increase in Th17 cells when compared to those cocultured with untreated macrophages (**Figure 5E**). Meanwhile, there was no statistical difference in the frequency of Th1 cells or Treg cells (**Figure 5E**). Macrophages might mainly affect type 17 immunity.

### 3.5 GM-CSF-promoted inflammatory macrophage differentiation associated with glycolysis-related gene upregulation

**Figure 6:**
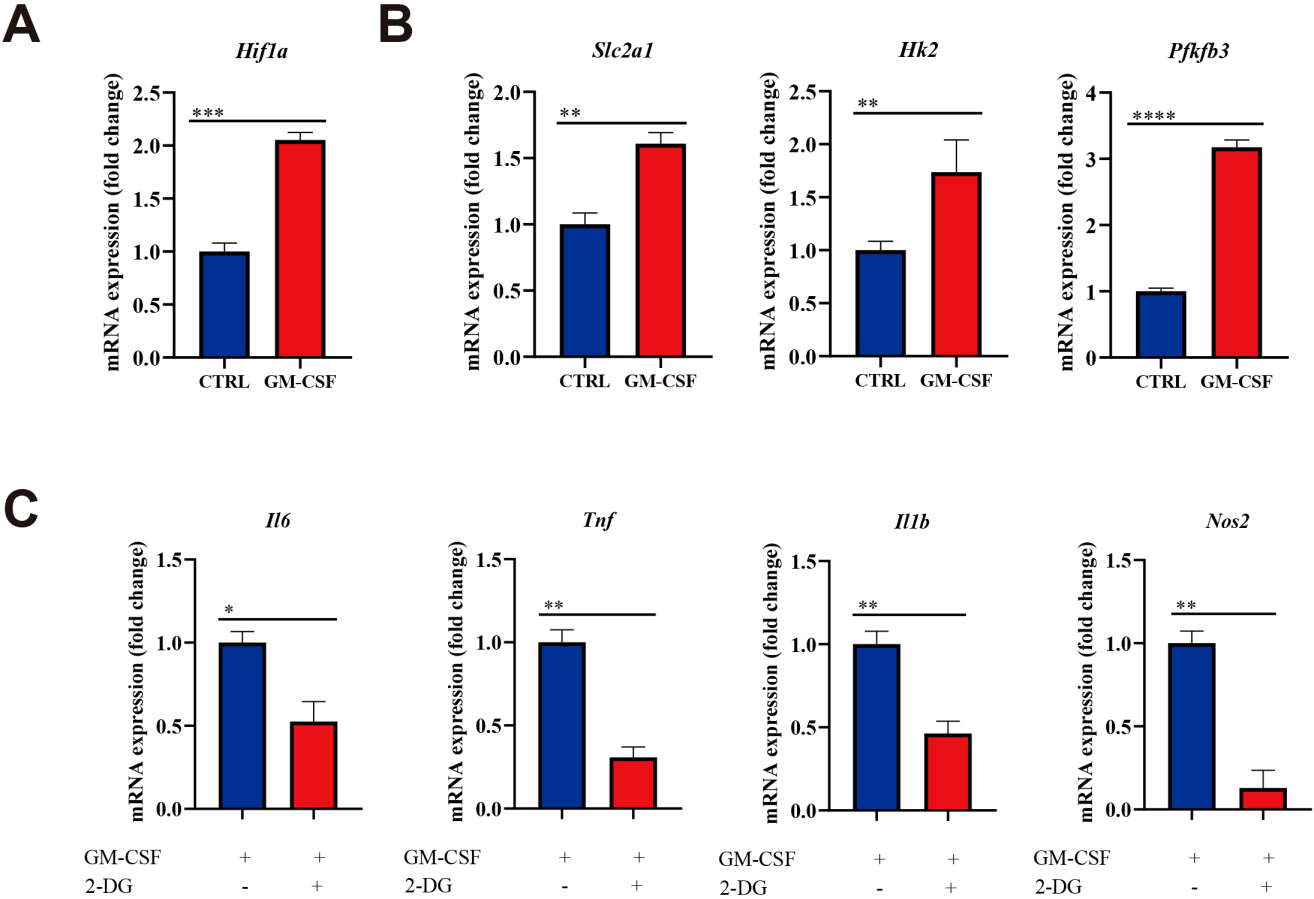
Macrophage activation in the presence of GM-CSF was associated with upregulation of glycolysis-related genes. **(A)** The mRNA expressions of *Hif1a* in peritoneal macrophages without and with rm GM-CSF treatment. **(B)** The mRNA expressions of *Slc2a1*, *Hk2*, and *Pfkfb3* in peritoneal macrophages without and with rm GM-CSF treatment. **(C)** The m RNA expressions of *Il6*, *Tnf*, *Il1b* and *Nos2* in GM-CSF-stimulated peritoneal macrophages without 2-DG treatment and with 2-DG treatment. * P< 0.05, ** P< 0.01, ***P< 0.001, ****P< 0.0001.

Functionally heterogeneous macrophages are shaped by various signals to adapt to their surroundings. Recently, emerging studies have pointed out that metabolic reprogramming has a marked effect on the phenotype and function of macrophages (58). Macrophages utilize oxidative phosphorylation (OXPHOS) to produce ATP under homeostatic conditions and reprogram metabolism to glycolysis during inflammation (58). It has been reported that M1 macrophages tend to mobilize glycolytic pathways, which leads to a large amount of glucose consumption and lactate production, while M2 macrophages exercise the oxidative phosphorylation pathway to utilize glucose (58). As shown above, GM-CSF showed proinflammatory ability, so we questioned whether GM-CSF affected the glycolysis of macrophages. It was reported that HIF-1α was a key transcription factor affecting the levels of glycolytic enzymes, and it was increased in M1 macrophages (59). We found that it was anabatic in GM-CSF-stimulated macrophages (**Figure 6A**). Moreover, glycolytic enzyme genes (*Slc2a1*, *Hk2*, and *Pfkfb3*) were also increased (**Figure 6B**). Then, we explored the association between glycolysis and macrophage polarization. Blocking the glycolytic pathway with the glycolytic inhibitor 2-deoxy-D-glucose (2-DG) suppressed GM-CSF-induced proinflammatory cytokine expressions (**Figure 6C**). Collectively, these results were suggestive of a trend toward glycolytic reprogramming in GM-CSF-stimulated macrophages, which might be associated with M1-like polarization.

### 3.6 GM-CSF regulated the Akt/mTOR pathway affecting glycolysis-related proinflammatory macrophage differentiation

**Figure 7:**
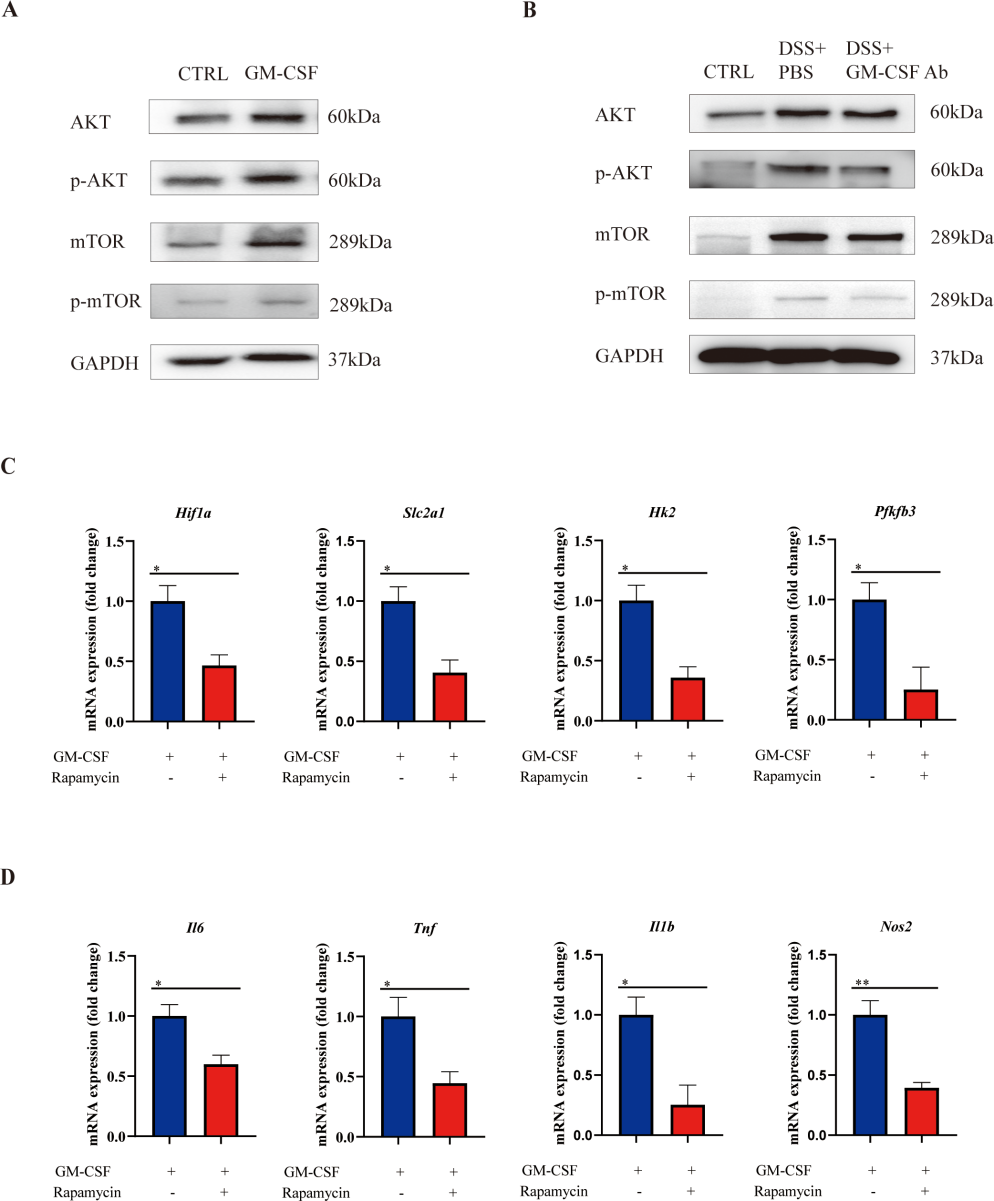
GM-CSF primed the Akt/mTOR pathway to regulate the expressions of glycolysis-related gense and then polarized macrophages. **(A)** Protein expression levels of Akt, mTOR, p-Akt and p-mTOR in peritoneal macrophages without and with rm GM-CSF treatment. **(B)** Protein expression levels of Akt, mTOR, p-Akt and p-mTOR in mice colonic tissues from the CTRL group, DSS+PBS group and DSS+GM-CSF Ab group. **(C, D)** The mRNA expression levels of *Hif1a*, *Slc2a1*, *Hk2*, *Pfkfb3, Il6*, *Tnf*, *Il1b*, and *Nos2* in rm GM-CFS-stimulated peritoneal macrophages without rapamycin treatment and with rapamycin treatment. * P< 0.05, **P< 0.01.

Akt and mTOR are important modulators of cell metabolism (60, 61). Studies have suggested that HIF-1α is an important downstream molecule of Akt/mTOR signaling in intercellular metabolism (62, 63). Thus, it was verified whether GM-CSF regulated the Akt/mTOR pathway. rm GM-CSF was used to stimulate peritoneal macrophages, and then the expressions of Akt, p-Akt, mTOR and p-mTOR were detected. It was shown that the phosphorylation of Akt and mTOR was raised, indicating that Akt and mTOR were activated (**Figure 7A**). In accordance with the results of cell experiments, DSS treatment led to the phosphorylation of Akt and mTOR, but GM-CSF Ab inhibited the phosphorylation in the intestines of colitis mice (**Figure 7B**). When Akt/mTOR signaling was obstructed by rapamycin, the capacity of GM-CSF to upregulate glycolysis-associated genes and proinflammatory genes was restrained (**Figures 7C, D**). These results implied that GM-CSF modulated macrophage glycolytic metabolism and polarization, potentially involving the Akt/mTOR pathway.

## 4 Discussion

Our study confirmed the high expression of GM-CSF in the inflamed colon of patients with UC and found that GM-CSF Ab alleviated DSS-induced colitis in mice. Then, we probed the possible mechanism of GM-CSF in colitis. The results showed that GM-CSF altered macrophage inflammatory phenotypes, as evidenced by enhanced expression of M1-associated markers, and augmented type 17 immunity during inflammation. Our results implied that GM-CSF modulated the function of macrophages to promote inflammation through the Akt/mTOR-dependent glycolysis pathway.

Numerous studies disclosed that GM-CSF or GM-CSF receptor was ascended and related to disease severity in many inflammatory or autoimmune diseases (64, 65). Many recent preclinical studies have revealed the therapeutic potential of targeting GM-CSF or GM-CSF receptor (32, 37, 38). Our study found that GM-CSF was elevated in colon tissues of UC patients. This result was consistent with a previous study (66). Several limitations of this study should be noted. The sample size was relatively small, and correlation analyses between GM-CSF expression and clinical parameters (e.g., disease severity, inflammatory markers) were not performed. However, these limitations do not affect the role of this finding as a “clue” for our subsequent mechanistic studies. Future studies with larger cohorts may further explore the clinical relevance of GM-CSF in UC.

GM-CSF can be secreted by diverse hematopoietic and nonhematopoietic cells, but the significance of each individual subset to produce GM-CSF in different diseases remains unclear (67). In experimental autoimmune encephalomyelitis (EAE), Th cells producing GM-CSF are related to the occurrence and development of diseases (68, 69). In serum transfer-induced arthritis, NK cells serve as a dominant source of GM-CSF and contribute greatly to disease progression (70). Regarding intestinal inflammation, several studies have demonstrated that GM-CSF is mainly produced by ILCs and orchestrates the development of disease (43, 56). Herein, we sought to examine the spatial distribution of GM-CSF within intestinal tissues during inflammation, as a first step toward understanding which compartments might contribute to increased GM-CSF production. Using immunofluorescence staining, we observed that GM-CSF expression was elevated in both the epithelial layer and the lamina propria of UC tissues compared to normal controls. However, we did not perform co-localization analyses with cell-specific markers (e.g., EpCAM for epithelial cells, CD4 for T cells, CD68 for macrophages, or CD127 for ILCs). Therefore, we cannot further determine the specific cell types responsible for the increased GM-CSF expression. Our findings are limited to describing the spatial distribution pattern of GM-CSF, and the exact cellular sources of GM-CSF in inflamed UC tissues remain to be clarified in future studies using multi-parameter co-staining approaches.

Previous studies on the significance of GM-CSF in gut inflammation seemed controversial (29). The contested data of animal experiments on GM-CSF functions in intestinal inflammation may partly be attributed to differences in experimental conditions, such as disease stage, cellular source and target cell type, model differences, as well as GM-CSF concentration and exposure duration. In our study, we found that GM-CSF acted on pathopoiesis in DSS-induced acute colitis. Administration of GM-CSF Ab to block GM-CSF in DSS-induced colitis mice ameliorated inflammatory manifestations with less weight loss, lower DAI and lower histology scores of colitis. Myeloid cells, especially macrophages, are the central target cells of GM-CSF (56, 71). Changes in macrophage infiltration in the colon were detected, and GM-CSF Ab visually decreased the infiltration of macrophages in the colon of DSS-treated mice. In addition, GM-CSF Ab balanced the responses of M1/M2 macrophages. The UC or UC animal model is characterized by disturbed immune responses of inflammatory Th cells (72, 73). The frequencies of Th cells were detected by flow cytometry in our animal experiment, and GM-CSF Ab treatment suppressed the excessive inflammatory Th cell responses, such as Th1 and Th17 cells. Under the experimental conditions of this study, these results indicated that GM-CSF disrupted intestinal immune homeostasis, boosting proinflammatory macrophage polarization and enhancing the responses of proinflammatory Th cells. While this study utilized antibody blockade as a classic intervention strategy to neutralize GM-CSF in colitis, future adoption of complementary approaches—including recombinant GM-CSF administration, conditional knockout models, or receptor-specific targeting—would help strengthen the causal interpretation. Furthermore, given that GM-CSF may play context-dependent roles, further studies are warranted to elucidate its distinct functions in intestinal inflammation and repair.

Macrophages play multifarious roles in maintaining gut homeostasis, but they are strikingly altered in UC (74). Increasing evidence supports the benefits of taking macrophages as a therapeutic target in UC treatment (75, 76). GM-CSF is a powerful regulator of macrophages. Despite being predominantly deemed a proinflammatory factor for macrophages, studies have suggested that GM-CSF is also related to M2-like macrophages (77, 78). It was assumed that this difference might be related to the dose and source of GM-CSF (67, 79). Moreover, recent studies have pointed out the importance of tissue “niches” in imprinting tissue-specific characteristics for macrophages, and different macrophage subsets might show different responses to the same irritants (80–82). Thus, to better characterize the effect of GM-CSF on intestinal macrophages, peritoneal macrophages, which were adjacent to the gut macrophage niches, were used as tool cells. We proved the proinflammatory effect of GM-CSF on peritoneal macrophages *in vitro*, as it stimulated the expression of inflammatory cytokines and M1-specific proteins. Macrophages regulate the immune network during intestinal inflammation through intercellular interactions. *In vitro* experiments showed that macrophages treated with GM-CSF enhanced the immune response of Th17 cells. These results coincided with the function of GM-CSF verified by Tomas Castro-Dopico et al. in mice (56). The results indicated the influence of GM-CSF on M1 macrophage polarization and type 17 immunity modulation in the intestine during colitis.

While our data provide evidence for GM-CSF-driven M1-like polarization, several open questions remain. Specifically, whether GM-CSF modulates other, less canonical macrophage activation states in the complex intestinal microenvironment is not yet known. In this study, we employed a classic panel of M1/M2-associated markers (NOS2, IL-6, TNF-α, IL-1β, CD206, IL-10, and Arg1); however, macrophage activation *in vivo* is likely overlapping and dynamic. Although peritoneal macrophages share certain similarities with intestinal macrophages, differences in phenotype, metabolism, and function still exist between peritoneal macrophages and resident intestinal macrophages. In future experiments, the use of primary intestinal macrophages and macrophage-organoid co-culture systems may serve as complementary approaches for further validation. Future studies incorporating a broader array of surface and intracellular markers, or single-cell transcriptomic approaches, will be important to fully characterize the spectrum of GM-CSF-induced macrophage phenotypes in the inflamed gut. The present study showed that GM-CSF-treated macrophages could enhance Th17 responses, although the precise mechanisms require further investigation in future experiments. Additionally, GM-CSF acts on multiple myeloid cell populations, and macrophage-specific intervention experiments *in vivo* were not performed in this study. Therefore, the contribution of other myeloid cell types cannot be excluded. Future studies using macrophage-specific GM-CSFR knockout, macrophage depletion, or adoptive transfer experiments are needed to further validate the specific role of macrophages in GM-CSF-mediated colonic inflammation.

Emerging evidence argues that metabolic reprogramming is crucial for macrophage activation during immune responses (83–86). Commonly, M1 macrophages are characterized by aerobic glycolysis (87). Glycolysis is the process of using glucose to produce energy and lactic acid through a series of enzymatic reactions. To explore whether GM-CSF was involved in metabolic reprogramming of macrophages, the mRNA transcription of glycolytic enzymes was scrutinized. Similar to the results in murine bone marrow-derived macrophages (BMDMs) and human peripheral blood macrophages, our study found that GM-CSF accreted the transcript levels of glycolysis genes (*Slc2a1*, *Hk2*, and *Pfkfb3*) in peritoneal macrophages (88, 89). This observation was suggestive of a potential metabolic reprogramming, which may contribute to macrophage inflammatory activities, as treatment with the glycolytic inhibitor 2-DG attenuated the expression of GM-CSF-induced proinflammatory genes. As HIF-1α was identified as a key regulator of glycolytic enzyme genes such as *Slc2a1*, *Hk2*, and *Pfkfb3*, its mRNA level was detected, and GM-CSF treatment increased the expression of *Hif1a (*90). Therefore, the current gene expression data were suggestive of glycolytic reprogramming. While direct measurements of glycolytic flux (e.g., ECAR, glucose uptake, or lactate production) were not performed in the current study, we plan to incorporate these analyses in future investigations to provide more definitive evidence of GM-CSF-induced glycolytic reprogramming. Moreover, future experiments involving genetic ablation of key glycolytic enzymes or the application of more specific inhibitors are warranted to further establish a causal relationship.

The contribution of the Akt pathway in modulating survival, proliferation, activation, migration and masterminding the response to metabolism has been widely recognized (91, 92). The Akt pathway can be activated by cytokines, pathogen recognition, and other stimuli, which can stimulate mTOR activity later (91). The serine threonine kinase mTOR is a conserved protein in mammals and plays a major role in cell metabolism (93, 94). In addition, the activation of mTOR has been reported to enhance the expression of HIF-1α to promote glycolysis (62). Considering that Akt and mTOR are important nodes in the metabolic reprogramming of macrophages, the Akt/mTOR pathway has been checked to determine whether it is downstream of GM-CSF in macrophages. Our study showed that GM-CSF triggered the Akt/mTOR pathway in peritoneal macrophages. This finding was also confirmed in GM-CSF Ab-treated DSS mice. Meanwhile, rapamycin, an inhibitor of mTOR, hindered the elevation of glycolysis-related genes induced by GM-CSF. Likewise, rapamycin prevented GM-CSF from promoting the expression of inflammation-related genes. Our data indicated that macrophages would differentiate toward a proinflammatory phenotype via Akt/mTOR-associated glycolysis in the presence of GM-CSF. In future studies, additional experiments using Akt inhibitors or genetic approaches (such as knockout or knockdown of Akt or mTOR) would substantially strengthen the mechanistic conclusions.

In conclusion, this study suggested that GM-CSF was increased in the intestine during inflammation, and that GM-CSF was increased both in the mucosal epithelium and lamina propria. Our results indicated that GM-CSF induced the phosphorylation of Akt and mTOR in macrophages to modulate the upregulation of glycolysis-related genes and then enhanced the pro-inflammatory characteristics of macrophages, which was associated with enhanced Th17 responses. These findings raise the possibility that targeting the GM-CSF/Akt/mTOR/glycolysis axis to modulate GM-CSF-mediated macrophage activation during intestinal inflammation may represent a potential direction for future research in UC, although further translational studies and long-term safety evaluations are needed.

## Data Availability

The raw data supporting the conclusions of this article will be made available by the authors, without undue reservation.
