# Supplementary material for: GM-CSF promotes pro-inflammatory macrophage activation associated with Akt/mTOR signaling during experimental colitis
Source: Front Immunol. 2026 Jun 26;17:1799536. doi: 10.3389/fimmu.2026.1799536 (PMC13349760; doi:10.3389/fimmu.2026.1799536)
Supplement: Supplementary file 1 [file DataSheet1.pdf]

frontiers

The International Society for the Advancement of Cytometry (ISAC) has highlighted the importance of including comprehensive methodological information to ensure data reproducibility and reliability. In line with this, Frontiers in Immunology now requires authors to submit a checklist for manuscripts that involve flow or mass cytometry. This checklist helps standardize the reporting process, improving the quality and transparency of published data. By doing so, we support scientific progress, making it easier for other researchers to replicate and validate experiments.

This form should be submitted with any manuscripts using flow or mass cytometry.

Sample/specimen/material description

☐ Total blood

☐ PBMCs

☑ Organ digests

Other____

Did the samples suffer any treatment before or after incubation with the antibodies?

☐ Drug ____________________________________

☑ Cell permeabilization _Foxp3 staining kit (00-5523-00, Thermo Fisher)_________

☑ Dye _Fixable Viability Stain 780 (565388, BD)

☐ Propidium iodine

☐ Not applicable

Other _Leuko Act Cktl with GolgiPlug (550583, BD),Ms CD16/CD32 (553141, BD)_______

Instrument and antibodies

Name of the Cytometer____FACSAria SORP (BD, USA)______________________________

Antibodies and targets	Fluorochrome/ Metal	Catalog number/Company
e.g. anti-CD4	FITC	Cat. XXX/ XXX ltd.
anti-Ms CD45	Alexa 700	560510, BD

anti-Ms CD3e	BUV395	563565, BD
anti-Ms CD4	BV510	563106, BD
anti-Ms IFN-Gma	BV711	564336, BD
anti-Ms IL-17A	BV421	563354, BD
anti-Ms Foxp3	PE	560408, BD

Data analyses

1. Name of the software____FlowJo software (version 10)________________________
2. Reference gating strategy in the manuscript or supplementary material

Gating strategy in (eg Figure X) __Materials and Methods
