# Supplementary material for: GM-CSF promotes pro-inflammatory macrophage activation associated with Akt/mTOR signaling during experimental colitis
Source: Front Immunol. 2026 Jun 26;17:1799536. doi: 10.3389/fimmu.2026.1799536 (PMC13349760; doi:10.3389/fimmu.2026.1799536)
Supplement: Supplementary file 2 [file SupplementaryFile1.docx]

**Supplementary Text S1. Flow cytometry gating strategies.**

**Gating strategy for macrophage subsets in LPMCs**

Gating was performed sequentially. Debris and aggregates were first excluded on an FSC-A vs. SSC-A plot. Viable singlet cells were then selected by Fixable Viability Stain 780 staining versus FSC-H. CD45⁺ cells were subsequently isolated, followed by identification of CD11b⁺ cells within the CD45⁺ population. Finally, F4/80⁺ cells were gated from the CD11b⁺ population.

**Gating strategy for helper T cell subsets (Th1, Th17, Treg) in LPMCs**

Gating was performed sequentially. Debris and aggregates were first excluded on an FSC-A vs. SSC-A plot. Viable cells were then selected by Fixable Viability Stain 780 staining versus FSC-H. CD45⁺ cells were subsequently isolated, followed by identification of CD3⁺ cells within the CD45⁺ population. CD4⁺ T cells were then gated from the CD3⁺ population. Finally, Th1 (IFN-γ⁺), Th17 (IL-17⁺), and Treg (Foxp3⁺) subsets were gated from the CD4⁺ population.
